# Historical Photogrammetry: Bird's Paluxy River Dinosaur Chase Sequence Digitally Reconstructed as It Was prior to Excavation 70 Years Ago

**DOI:** 10.1371/journal.pone.0093247

**Published:** 2014-04-02

**Authors:** Peter L. Falkingham, Karl T. Bates, James O. Farlow

**Affiliations:** 1 Structure and Motion Laboratory, Department of Comparative Biomedical Sciences, Royal Veterinary College, London, United Kingdom; 2 Department of Ecology and Evolutionary Biology, Brown University, Providence, Rhode Island, United States of America; 3 Department of Musculoskeletal Biology II, Institute of Ageing and Chronic Disease, University of Liverpool, Liverpool, United Kingdom; 4 Department of Geosciences, Indiana-Purdue University, Fort Wayne, Indiana, United States of America; University of Pennsylvania, United States of America

## Abstract

It is inevitable that some important specimens will become lost or damaged over time, conservation is therefore of vital importance. The Paluxy River dinosaur tracksite is among the most famous in the world. In 1940, Roland T. Bird described and excavated a portion of the site containing associated theropod and sauropod trackways. This excavated trackway was split up and housed in different institutions, and during the process a portion was lost or destroyed. We applied photogrammetric techniques to photographs taken by Bird over 70 years ago, before the trackway was removed, to digitally reconstruct the site as it was prior to excavation. The 3D digital model offers the opportunity to corroborate maps drawn by R.T. Bird when the tracksite was first described. More broadly, this work demonstrates the exciting potential for digitally recreating palaeontological, geological, or archaeological specimens that have been lost to science, but for which photographic documentation exists.

## Introduction

The fossil footprints and trackways preserved in the Glen Rose Formation along the Paluxy River at Glen Rose, Texas (USA) are among the most famous dinosaur tracks in the world, and represent a colossal wealth of information about dinosaur palaeobiology, being exceptionally well preserved and extremely extensive. Although tracks from the Paluxy River were published on as early as 1917 [1]–, it was Roland T. Bird, a fossil collector for Barnum Brown of New York's American Museum of Natural History (AMNH), who recognised unambiguous sauropod tracks for the first time [4], tracks which would later be designated the holotype of *Brontopodus birdi* [5]. Among the many diverse trackways that Bird examined in the Glen Rose Formation sites across Texas, were sauropod manus-only trackways, interpreted by Bird to have been produced by a swimming sauropod [6], [7]. But it is perhaps the Paluxy River's ‘chase sequence’ that is the most infamous from the area. In 1940 Bird returned to Texas on an expedition to collect portions of this ‘chase sequence’ in which a theropod was apparently following a sauropod [6], [8], [9].

The section of trackway collected measured over 9 m in length and 3.65 m in width, and in order to remove the tracks, the entire section was broken into large blocks [10]. The blocks were then transported to separate locations – one part of the trackway was housed at the Texas Memorial Museum (TMM), and one part was sent to the AMNH. The final portion of the trackway has since been lost or destroyed. The trackways remain on display at their respective museums. However, in 1988 deterioration of the trackway section held at the TMM was reported, and that deterioration has continued since [11].

Given the significance of the tracks located in the Paluxy River, as well as the TMM and AMNH, and because the tracks are subject to the destructive forces of said river, it is imperative that on-going efforts are made to document the tracks in as accurate and systematic way as possible. Since 2008 a team has been doing just that, using overhead photography, LiDAR laser scanning, photogrammetry, and other techniques [8], [11].

Of the methods employed in the documentation of the Paluxy River tracks, photogrammetry has seen phenomenal advancement in recent years, to the point where it has attained comparable accuracy and resolution to laser scanning [12]. Where once the generation of a 3D digital model required the transport and set up of a large, heavy, and expensive laser scanner on top of a large platform over the river (and where that laser scanner would often shut down due to the ambient heat), an accurate and high resolution, phototextured, digital outcrop model (DOM) can now be made using photos from a consumer digital camera and free software [13].

While the application of photogrammetry to the site has considerably increased the ease with which modern documentation can be carried out, it has also raised the exciting opportunity to retrospectively produce digital models from photographs. Much as the significance of the Paluxy River tracks has led to a major documentation effort today, so too did R.T. Bird ensure that efforts were made to record the ‘chase sequence’ prior to, and during, excavation. To this end, R.T. Bird took numerous photographs, as well as a film, during the excavation in 1940, to complement his hand-drawn cartographic maps.

Because modern photogrammetric software is able to account for unknown focal lengths and camera types, the photos taken by Bird over 70 years ago can be used to generate a digital model of the tracks as they were in 1940, *in situ* prior to excavation. In this paper we have taken Bird's original photographs, and used them to generate a digital model of the Paluxy River ‘chase sequence’. This model is then compared with Bird's original map drawings, as well as with laser scan data of the TMM and AMNH blocks.

## Materials and Methods

**Figure 1:**
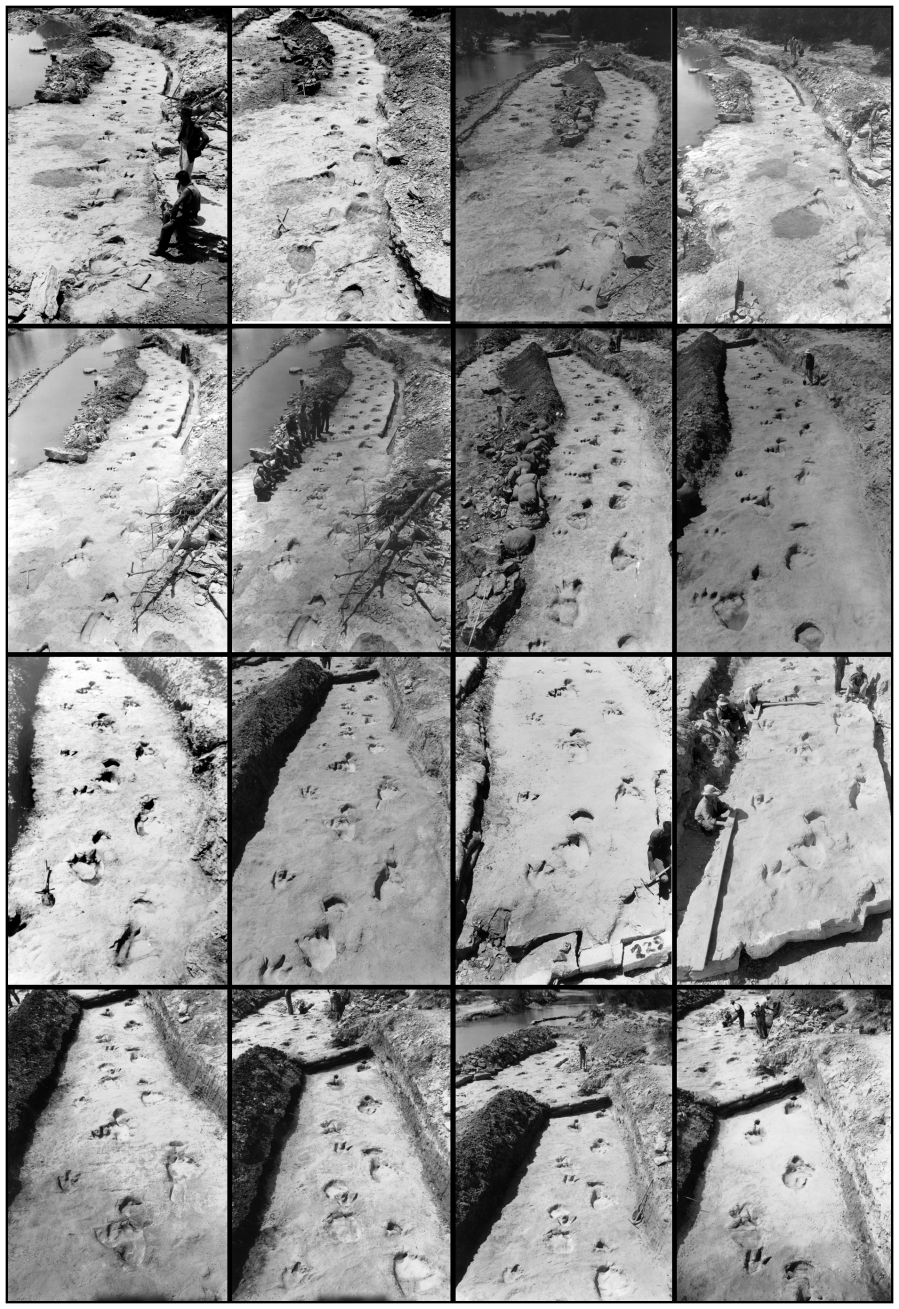
Sixteen of Bird's original photographs used in the photogrammetric reconstruction of the trackway. Note that the state of excavation (flooded parallel trackways, sandbags, tools etc) varies between images, causing complications for the reconstruction.

Seventeen photographs (either 5 inches by 7 inches, or 8 inches by 10 inches, in size) and/or negatives made by Bird during the excavation in 1940 were used (Figure 1). Unfortunately, the photographs come from a number of stages throughout the excavation process, and as such the content of the photographs varies, including images prior to and after damming/flooding of a parallel exposed trackway, the emplacement of sandbags, and the excavation itself, with one photograph recording the breakup of the trackway section. In addition, multiple shots contain people, or tools such as picks and shovels, which do not remain stationary between images. These factors are generally detrimental when producing photogrammetric models, and should always be avoided if possible when collecting data. Obviously in this case however, the data contain these complications and there is little can be done to remove them whilst retaining primary information. It is likely that these issues will be common to historical photogrammetry.

Bird's photographs or negatives were scanned at a resolution of 5436×7787 pixels (Figure 1). In order to process the photographs and produce a digital model, the freely available software VisualSFM [14]–[16] was used to match the photos and generate a sparse reconstruction. The difficulties associated with the photographs, listed above, meant that it was difficult for the software to match images; only 12 of the original 17 photographs could be matched to each other and used to generate a model.

This sparse point cloud was then processed using PMVS and CMVS (through the VisualSFM GUI) to generate a dense point cloud [17]–[19]. A polygonal surface was generated from the dense point cloud using the Screened Poisson Surface Reconstruction algorithm [20].

In addition to the digital model produced from photographs, previously collected laser scans of the TMM and AMNH sections of the trackway in their current conditions were used for comparative purposes. These models were produced using a Reigl LMS-Z420i LiDAR laser scanner during previous work [8], [11] and readers are referred to those publications for additional details. Because photogrammetry is an inherently scale-less method, scale must be applied after the photogrammetric model is generated. In order to do this, we used measurements between distinct tracks from both Bird's original maps and the laser scans in the areas that the photogrammetric model displayed highest fidelity.

**Figure 2:**
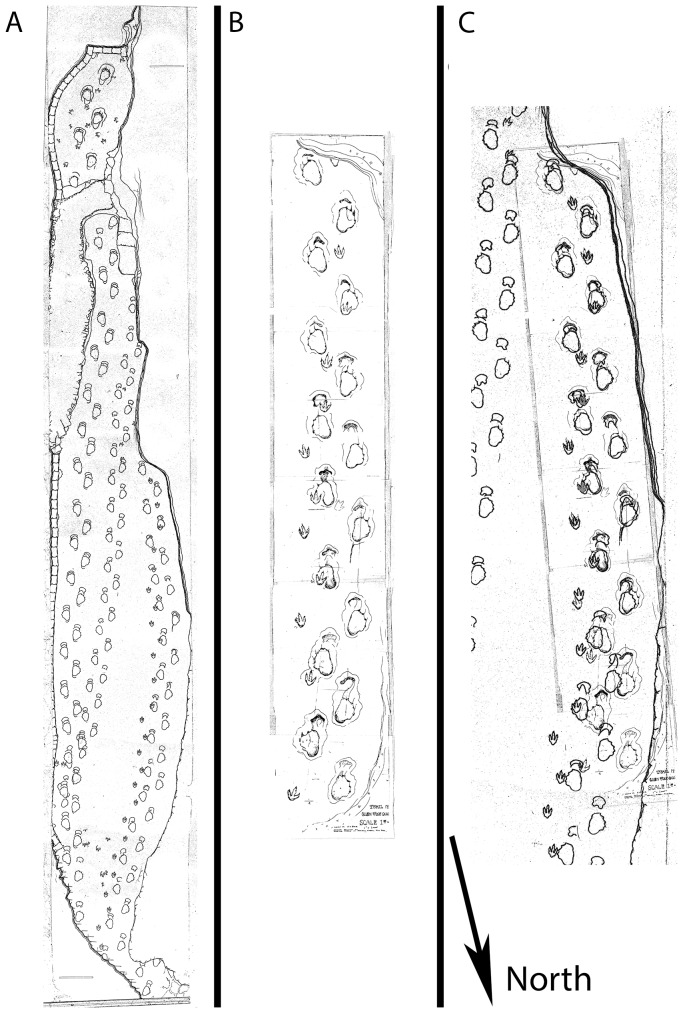
R.T. Bird's maps of the Paluxy ‘chase sequence.’ a) Bird's Rye chart, b) the Austin chart, and c) the Austin and Rye charts overlaid. Note that the Austin and Rye charts diverge toward the north.

In order to compare and corroborate the photogrammetric reconstruction, two maps drawn by R.T. Bird were used – the Austin chart and the Rye chart (Figure 2) [5]. These maps were drawn by R.T. Bird around the time the trackways were excavated. However, the drawings are not identical – in the Austin chart the trackways are much straighter, while in the Rye chart the trackways curve gently to the left (Figure 2c).

## Results

**Figure 3:**
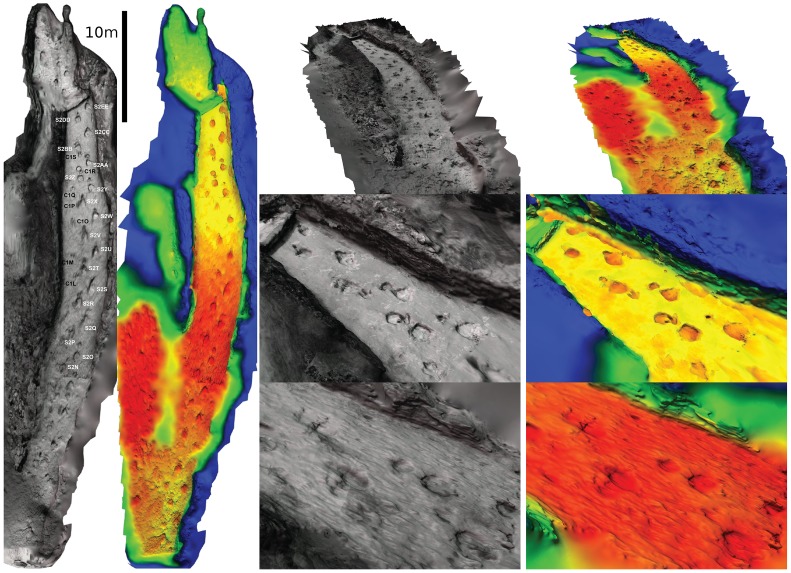
Photogrammetric reconstruction of Bird's chase sequence. Far left, photo-textured and height mapped plan-view of the reconstructed trackway. Track labels according to Farlow et al. (1989). Right, photo-textured and height mapped views, top to bottom; isometric view along trackway, close up of high fidelity southern end, close up of poor quality northern end.

The nature of the photographs - low sharpness, mobile features (people, tools) over the site, and the fact that the photographs were all taken from a roughly south facing direction - meant that parts of the resulting model, particularly at the northern end, are severely lacking in detail. In addition, the single general direction of the photographs created linear artefacts running along the length of the reconstructed model. Nevertheless, the entire sequence observable from the photographs was reconstructed in 3D, measuring over 45 m in length. Although the tracks lack fine detail, their locations are obvious on the textured model, and as such an aerial-view of the site, as it was prior to excavation, can be produced (Figure 3, Movie S1). In addition, we were able to replace the phototexture with colour corresponding to height, which accentuates the tracks, and aids in visually locating individual footprints.

In contrast to the northern and central portions of the trackway, the southernmost end, where 6 photographs are clustered (see Figure 1), has been reconstructed in enough detail to observe digit impressions in the theropod and sauropod tracks. This makes comparisons with R.T. Bird's original maps possible. By superimposing the digital reconstruction and the maps, it can be seen that the Rye chart, in which the theropod and sauropod trackways curve to the left, is a much better match than the Austin chart in which the trackways are much straighter.

## Discussion

The digital reconstruction shows high variations in quality along the length of the trackways. Those tracks in the middle section in particular are of poor quality. This portion of the reconstruction suffers because it is captured only in the upper, distant portion of half of the photographs; the remaining images do not show this portion of the trackway at all. However, the distal most portion of the trackway, near to the sandbag barrier, is of excellent quality (Figure 3, Supplementary data), with individual digit impressions of the theropod tracks visible, and variations in the base of the sauropod tracks observable in the height-mapped colouration. This shows that even with poor source photographs, highly detailed reconstructions are at least partially possible.

**Figure 4:**
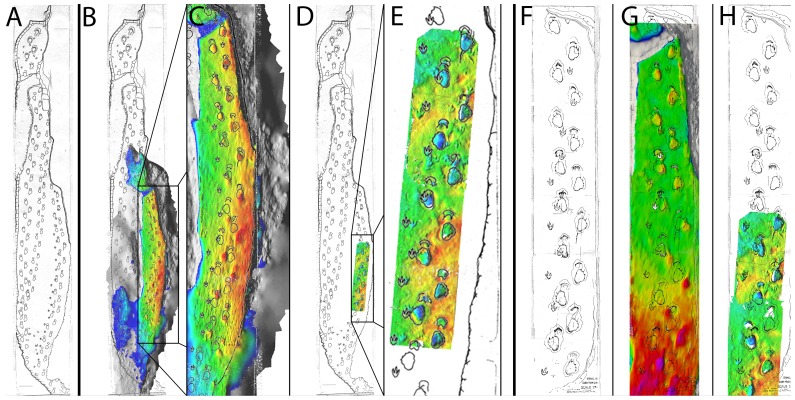
Overlays of Bird's Rye and Austin charts with photogrammetric and laser scan digital models. a) The Rye chart, b) portion of the Rye chart reconstructed via photogrammetry with historical photos, c) Close-up of photogrammetric reconstruction, d) location of laser scans of the AMNH and TMM sections in the Rye chart, e) close-up of match between Rye chart and laser scans, f) Austin chart, g) Austin chart and photogrammetric reconstruction, h) Austin chart and laser scans of AMNH and TMM sections.

When R.T. Bird drew his maps of the Paluxy trackways, he did so using lengths of string – fighting against constant flooding from the river [5]. It is unclear as to the timing of the creation of the Austin and Rye charts, but by comparing the maps with the photogrammetric reconstruction (Figure 4), it is clear that the Rye chart is the more accurate representation of the tracks as they were prior to excavation – the trackways curve gently to the left in both the Rye chart and the photogrammetric model, whereas they are straight in the Austin chart.

The laser scans of the AMNH and TMM sections indicate a number of theropod tracks that are not recorded in either of Bird's maps (Figure 4e, h), though these occur in an area of the photogrammetric model that is poorly resolved. If the charts and the photogrammetric model or laser scans are aligned based on the locations of the sauropod tracks, there is a slight offset to many of the theropod tracks. This may indicate that R.T. Bird measured and mapped the trackways independently of each other.

## Conclusions

Using a world-famous dinosaur tracksite, this work has demonstrated the potential for historical photogrammetry: the reconstruction of 3D digital models of specimens, sites, or exposures which have deteriorated, or been lost to science entirely, but are recorded with photographs taken prior to loss/deterioration. In this case, Bird's ‘chase sequence’ has not existed in a complete form since it was excavated in 1940 over 70 years ago. After excavation, some parts were distributed to several institutions, and others were lost completely. With the photogrammetric reconstruction, we were able to corroborate which of two differing maps of the trackway, drawn by R. T. Bird was the most accurate.

It is an exciting prospect to think that many palaeontological or archaeological specimens that have been lost to science, or suffered irreparable damage, may be digitally reconstructed in 3D using free software and a desktop computer. We envisage that historical photogrammetry will become a powerful, common, tool in the future, particularly as advances in photogrammetric techniques enable software to compensate for the difficulties inherent in using old photographs.

## Supporting Information

Movie S1 **Fly through of the reconstructed trackway.**
(AVI)
